# Model for Mitigation of Workplace Transmission of COVID-19 Through Population-Based Testing and Surveillance

**DOI:** 10.1089/pop.2020.0322

**Published:** 2021-02-05

**Authors:** Peter J. Plantes, Maren S. Fragala, Charles Clarke, Zachary N. Goldberg, Jeff Radcliff, Steven E. Goldberg

**Affiliations:** ^1^ hc1, Indianapolis, Indiana, USA.; ^2^ Quest Diagnostics, Secaucus, New Jersey, USA.

**Keywords:** COVID-19, population-based testing, SARS-CoV-2, surveillance, workplace mitigation

## Abstract

The coronavirus disease-2019 (COVID-19) pandemic is having a widespread impact on societies across the globe. As part of the effort to control transmission in the United States, many businesses either closed or instituted nonpharmaceutical control measures and allowed only essential workers on-site. During summer and fall of 2020, employers began formulating “return to work” strategies designed to mitigate the risk of transmission among employees. On a population level, several countries implemented national testing and surveillance strategies that proved effective in mitigating citizen-to-citizen transmission and contributed to suppressing COVID-19. A crucial component of many such strategies is population-based testing to identify and engage individuals with asymptomatic or presymptomatic infection, which also is relevant to return-to-work strategies. The authors describe an approach that multisite employers might use to help mitigate transmission of COVID-19 in the workplace. This approach leverages a bioinformatics platform informed by real-time PCR test data at the county and subcounty (eg, Public Use Microdata Area) level, allowing for population-based testing to be selectively targeted for employees in geographies with elevated SARS-CoV-2 positivity. A “Command Center” application integrates data from multiple sources (eg, local infection trends, employee symptom diaries, Bluetooth thermometers) in real time, which can be used to inform decisions regarding surveillance and employee self-isolation or quarantine; a mobile phone-based application provides for rapid, secure communication with employees. This overview is based on peer-reviewed literature and the early experience of a large employer with implementing bioinformatics tools to mitigate the impact of the pandemic on the workplace.

## Introduction

As the coronavirus disease-2019 (COVID-19) pandemic persists, employers, schools, and communities continue to focus on mitigating further spread of severe acute respiratory syndrome-coronavirus-2 (SARS-CoV-2). Employers are particularly focused on delivering a disciplined, systematic, and effective approach to employee safety from SARS-CoV-2 exposure, in the context of regulatory requirements from the Occupational Safety and Health Administration (OSHA)^1^ and the US Equal Employment Opportunity Commission (EEOC)^2^ for enforcing workplace safety standards during the COVID-19 pandemic. However, a challenge in current population health surveillance data is the lag time in reporting results, which presents a major barrier to initiating appropriate public health actions.^3^ A population health surveillance system based on population testing and real-time tracking may aid in filling this gap, helping to reduce transmission and provide a safe workplace during the pandemic. In a companion paper, Fragala et al.^4^ inventory the range of individual, public health, and workplace strategies that are being leveraged to facilitate return to work and mitigate workplace transmission. This paper (1) provides a brief overview of transmission dynamics; (2) describes tools that can be used to ascertain local risk for SARS-CoV-2 infection; and (3) outlines an integrated population health surveillance approach, based on local risk data, designed to help multisite employers mitigate worker-to-worker transmission and inform workplace decisions for employers as “action alerts.”

## SARS-CoV-2 Transmission

An understanding of characteristics that affect viral transmission of SARS-CoV-2 underlies strategies to mitigate or suppress transmission, whether in the workplace or community. Transmission of viral infections commonly occurs in households, schools, workplaces, and the community.^5,6^ Despite strong efforts to suppress and mitigate the spread of COVID-19, a retrospective cohort study from China early during the pandemic found that close contacts frequently become infected.^7^ Household contacts and those traveling with an active case had a 6- to 7-fold higher risk of contracting infection than other close contacts.^7^

Active SARS-CoV-2 is found and replicates primarily in the nasopharynx (upper respiratory tract) and oropharynx, including the throat and lungs.^8^ Symptoms develop 2–14 days after exposure,^9,10^ most often 4–7 days after exposure.^10,11^ High viral shedding from the throat (pharyngeal region) occurs during the first week of symptoms, with a peak on day 4.^8^ Shedding of replication-competent SARS-CoV-2 is believed to occur for up to 10 days following symptom onset in persons with mild to moderate COVID-19.^9^ Patients with severe COVID-19, including those who are immunocompromised, may shed replication-competent virus for up to 20 days.^9^ Viral ribonucleic acid (RNA) shedding (replication and release of viral particles) from saliva and mucus also continues beyond the end of symptoms.^8^ RNA may be detected for 24 days after symptom onset, with 10% remaining positive even 33 days after symptom onset.^8,12^

Importantly, viral shedding and transmission can happen even in the absence of symptoms. Presymptomatic and asymptomatic transmission can account for approximately 40%–45% of SARS-CoV-2 infections.^13,14^ Moreover, asymptomatic persons may transmit the virus to others for an extended period, perhaps longer than 14 days.^14–16^ Because of the high risk for silent spread by asymptomatic and presymptomatic persons, it is imperative that screening programs include testing of those without symptoms.^13,14^

### Workplace transmission

The workplace is an important source of potential transmission and, as such, can play a crucial role in containing the spread of an infectious disease outbreak.^17,18^ Most (75%) US workers are employed in occupations that cannot be done at home. The continuing need for health care, manufacturing, retail, and food services puts approximately 108.4 million workers at increased risk for adverse health outcomes related to working during a pandemic.^19^ Most exposed workers are employed in health care sectors^20^; other occupational sectors with high proportions of exposed workers include protective services, office and administrative support occupations, education, community and social services, construction, meat and poultry packing, and maintenance.^19,21^

The degree of exposure also varies among occupations. For example, approximately 10% (14.4 M) of US workers face exposure to infection at least once per week,^22^ and approximately 18.4% (26.7 M) face such exposure at least once per month.^22^ Exposure is generally defined by close contact (ie, within 6 feet for a total of 15 minutes or more) with a person with COVID-19,^23^ and is more likely in the aforementioned occupations. Although SARS-CoV-2 has different transmission characteristics than influenza (eg, higher transmissibility, longer incubation period, asymptomatic transmission, prolonged viral shedding),^24^ data from studies of influenza can shed light on the potential role of the workplace in SARS-CoV-2 transmission. Evaluations of influenza epidemics have demonstrated the substantial contribution of the workplace to transmission.^25,26^ As many as 20%–25% of weekly contacts are made in the workplace, and modeling studies suggest that 9%–33% of influenza transmission occurs in the workplace.^25,26^ A modeling study also suggested that most (72%) of the workplace transmission that occurs during an influenza epidemic results from exposure to employees who go to work sick (presenteeism).^25^ During the ongoing COVID-19 pandemic, increases in absenteeism have been reported among occupational groups less able to avoid exposure to SARS-CoV-2.^27^ The concern about exposure to SARS-CoV-2 in the workplace points to the need for accurate, real-time assessment of transmission risk along with measures to mitigate the risk. Risk assessment tactics include surveillance in the workplace as well as in regions that employees commute from.

### Workplace surveillance

Given the proportion of the US workforce exposed to infection at work, the workplace is a key focus for surveillance and public health interventions that could protect both workers and the communities they serve.^22,27^ Diagnostic testing, screening, and surveillance can help to monitor the burden of occupational exposure to infection and disease in a workforce, including how many workers are potentially exposed and what job functions and locations they work in.^22^ Population health surveillance helps to monitor and characterize a community for infection incidence and prevalence at a population level. Surveillance practices also allow for upstream prevention measures, both in terms of workplace practices (eg, plans, training) and policies (eg, sick leave, hazard pay).^22^

### Community surveillance

Countries have implemented different community surveillance and control measures. Community surveillance through tracking epidemiology and transmission among early cases in China^11^ and South Korea^28,29^ revealed that considerable efforts to reduce transmission are required to control outbreaks. Rapid testing and early identification enabled scaled-up measures for preventing community transmission (including isolation and contact tracing to reduce the time during which cases are infectious in the community, thereby reducing the spread)^7^ and facilitating treatment (including updated triage and treatment systems, mobilizing the necessary resources for clinical care, and mandatory mask wearing).^28,29^

### Nonpharmaceutical interventions to limit transmission

In the absence of a COVID-19 vaccine and proven treatments, nonpharmaceutical interventions aim to mitigate or suppress transmission of the virus by reducing contacts in the population.^5^ Mitigation aims to slow transmission. Mitigation efforts have the potential to reduce illness and mortality through tactics including home isolation of suspected cases, home quarantine of those living in the same household as suspect cases, contact tracing and isolation of individuals who may have been exposed to suspected cases, and social distancing.^5^ Suppression, on the other hand, aims to reverse epidemic growth, reducing case numbers to low levels and maintaining that situation indefinitely.^5^ Although suppression is preferable to mitigation from a public health perspective, the control measures needed to attain suppression may be impractical in some settings, such as essential industries.^5^ Employer programs can benefit from tools that facilitate deployment and integration of multiple strategies and surveillance tactics.

### Employers' duty to provide a safe working environment

OSHA requires employers to provide workers a working environment that is free from recognized hazards that cause or are likely to cause death or serious physical harm.^30,31^ Equal opportunity laws do not prevent employers from following the guidelines and suggestions from the Centers for Disease Control and Prevention (CDC) or state/local public health authorities about steps employers should take regarding COVID-19.^2^ Because the CDC and state/local health authorities have acknowledged community spread of COVID-19 and issued attendant precautions, employers may ask employees if they are experiencing symptoms (eg, fever, chills, cough, shortness of breath, sore throat) of the pandemic virus, and also measure employees' body temperature.^2^ COVID-19 testing is classified as a “medical test.” As such, the Americans with Disabilities Act (ADA) requires that any mandatory “medical test” of employees be “job related and consistent with business necessity.”^2^ Thus, as an individual with the virus will pose a direct threat to the health of others, employers may choose to administer COVID-19 testing to employees before they enter the workplace.^2^ Because the CDC states that employees who become ill with symptoms of COVID-19 should leave the workplace, the ADA does not interfere with employers following this advice.^2^ To prevent occupational exposure to SARS-CoV-2, OSHA offers employer guidance on infection prevention and industrial hygiene practices, focused on the need for employers to implement engineering, administrative, and work practice controls and personal protective equipment as well as considerations for doing so.^1^

## Workplace Programs

The rapid community spread of COVID-19 in the United States has created a need to mitigate the risk of transmission in the workplace. Careful planning is required to maximize the success of mitigation efforts, in terms of protecting employee privacy, employee acceptance, and program effectiveness. This section provides a brief overview of considerations employers may wish to address when deciding whether to implement programs to mitigate workplace transmission of COVID-19 and highlights important tools and processes that can facilitate such programs.

### Confidentiality of medical information in workplace programs

Employers must maintain all information about employee illness as a confidential medical record in compliance with the ADA.^2^ ADA requires that all medical information about a particular employee be stored separately from the employee's personnel file, thus limiting access to this confidential information.^2^ Thus, data sharing should be minimal with thoughtful justification and appropriate Health Insurance Portability and Accountability Act (HIPAA) authorization in place.

Within the workplace surveillance system, employee privacy can be controlled using strict role-based access and an affirmative consent model. Access to protected health information (PHI) and employee identifying data is limited to authorized human resources employees, tracked, and audited. Under the HIPAA Privacy Rule, an employer cannot access an employee's PHI without authorization unless other laws require them to do so. Each time an employee completes a symptom survey or receives a lab test, authorization is captured and sent to the system. Employees may revoke authorization at any time. Prior to displaying any employee's PHI, the system checks that a valid authorization exists.

### Planning a workplace testing program

Workplace testing programs can play an important role in reducing the risk of workplace transmission of SARS-CoV-2.^32^ The objectives of an employer population testing and surveillance program are similar to those of national or state programs–to mitigate transmission of SARS-CoV-2–but the focus is on the workplace(s) and on company employees. The local environment of an office building represents a work “microcommunity” that employees commute to each workday from various local towns and family units, each of which represents another microcommunity. To maintain a safe workplace, the employer needs to understand and track the risk factors that each employee may introduce into the worksite.

Workplace practices and policies can help to stop the spread of disease.^18,25,26^ For example, policies to prevent employees going to work sick have been shown to reduce workplace influenza infections by 25%–39%.^25^ Practices including workplace vaccination for COVID-19, when available, could play an important role in preventing an outbreak; this approach has shown efficacy for other infectious diseases.^18^

Implementation of tools and processes for COVID-19 surveillance in the workplace requires close engagement of several functions, including medical, compliance, regulatory, privacy, human resources, and legal teams. Key considerations include the opportunity for these functions to review information technology requirements and data flow documents to ensure compliance with privacy and HIPAA requirements. Quest Diagnostics implemented a workforce COVID-19 surveillance system (hc1 Workforce Advisor; hc1, Zionsville, IN) that was guided by an interdisciplinary steering committee under the direction of the company's Chief Health Officer, Health and Wellness, who serves as chair of the committee. The committee was delegated governance to formulate the company's pandemic engagement strategy. Committee recommendations are in turn presented to the Chief Human Resources Officer, who in turn engaged the company's senior management team for advice and consent on the strategic recommendations and authorization of needed actions and allocation of resources.

Despite the value of COVID-19 mitigation for the workforce, costs may present a barrier to implementation of a similar program for some employers. Implementation cost considerations include the costs of COVID-19 polymerase chain reaction (PCR) testing, software implementation and subscription charges for real-time data monitoring of symptoms, and capacity for contact tracing and hot spot monitoring. Another consideration is the potential impact of employees choosing not to participate in COVID-19 PCR testing. COVID-19 PCR testing is a voluntary component of the program, and lower opt-in rates will translate to fewer detected infections and fewer employees identified through contact tracing. This effect may be mitigated by using a variable testing strategy that focuses on communities with higher viral transmission, where asymptomatic viral shedding likely will be more common.

### Enhancing efficiency of population screening

Because SARS-CoV-2 can be transmitted during presymptomatic and asymptomatic infection,^13,14^ monitoring symptoms (eg, fever, cough, shortness of breath, fatigue) alone is insufficient to identify, track, and contain the spread. Mitigating or suppressing transmission requires extensive testing and data-driven decisions.^33,34^ Data derived from screening employee COVID-19 testing data and individual diagnostic data derived from the employer workplace protocol must arrive in a real-time, “alert-actionable” manner.

Determining the true prevalence in a population requires testing of randomly selected individuals from the community to ensure the estimates represent the population.^35^ As resources are constrained, population sampling with targeted “screening” protocols can help reduce the number of tests required for any given population.^35^ In addition, sample pooling has received emergency use authorization for clinical testing and offers another approach to facilitate surveillance by enabling expanded laboratory capacity.^36–38^

### Tracking SARS-CoV-2 positivity rates: percent positive

Tracking the SARS-CoV-2 test positivity, or percent positive, rate in a community can help to assess the level of transmission in the community and whether COVID-19 testing is sufficient. The percent positive is the percentage of all SARS-CoV-2 tests that have positive results: percent positive = (number of positive tests ÷ number of total tests) x 100%. A high percent positive rate can indicate that either transmission in the community is high or testing is insufficient, and that restrictions may be necessary to slow the spread of disease. As a guideline, the World Health Organization recommended in May that the percent positive remain below 5% for at least 2 weeks before governments consider reopening.^39^ However, changes in testing practices and capacity and delays in reporting can result in biased estimates and should be monitored and taken into consideration when interpreting COVID-19 infection rates.^40^

### Data surveillance system

The availability of timely data is essential to identify local transmission and take corrective actions to minimize spread of COVID-19 in the workplace. Inclusion of critical data from diagnostic testing, hospital case reports, serologic testing, community demographics, and contact tracing, which come from disparate sources, can be considered. Therefore, connecting distinct data systems and data among laboratories, health systems, and public health agencies is essential for disease surveillance and managing the crisis.^3,41^

Prompt and accurate laboratory testing and reporting enable communities to track disease trends, identify outbreaks, and diagnose health conditions.^42^ Electronic data transmission with standard processes and reporting formats facilitates prompt sharing of this critical information.^42^ Electronic laboratory reporting provides vital information from private and public health laboratories and hospitals on health conditions to local and state public health departments.^42^

Linking electronic laboratory test data to population demographic data enables transformative evaluation.^41^ US census data are publicly available as Public Use Microdata Areas (PUMAs), which provide geographic substate demographic data.^43^ PUMAs have been used in research^44^ and have helped catalyze knowledge generation across a wide range of social science and other disciplines.^44^ This data integration strategy facilitates population health surveillance by the geographic distribution of the population.^41^

### Tactics to help the employer discover infectious asymptomatic and presymptomatic infection

In the workforce COVID-19 surveillance program at Quest, laboratory test results (COVID-19 PCR test results) available at the county or PUMA level inform employer worksite actions to mitigate impacts of the pandemic. For example, data integration enables tracking and following the pandemic burden in the residential community of employees. In settings where transmission is expanding, the pandemic burden in each employee's residential community can be early indicators of pandemic burden and transmission risk with potential impact to the employee pool.

Workplace mitigation strategies require integration of testing data and surveillance data about the workplace and in communities where employees live. Thus, the following tactics may help employers identify and respond to changes in local transmission and help inform workplace interventions.

1. Population-based SARS-CoV-2 diagnostic testing of asymptomatic individuals

A systematic and disciplined approach to population-based COVID-19 viral testing (ie, viral antigen test, PCR test) can be implemented.^35^ Because of the high risk for silent spread by asymptomatic and presymptomatic persons, testing programs must include those without symptoms.^13,14^ A population testing program (at some frequency each week) of a designated population of regular commuters can be implemented at a company worksite, university campus, or other such facility.^35,45^

2. Monitoring trends in the pandemic burden of residential communities

**FIG. 1.**
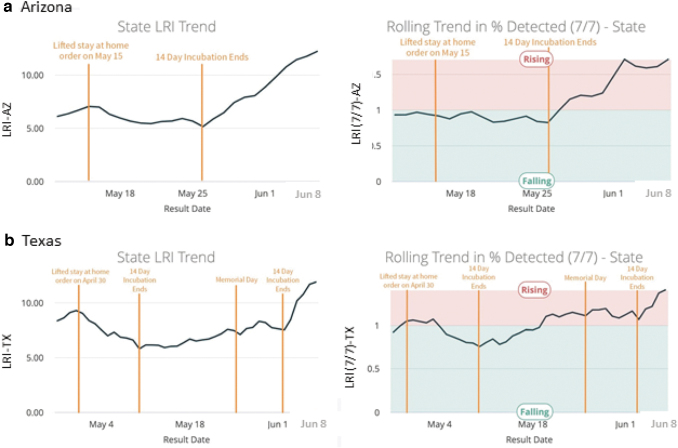
LRI trends for **(a)** Arizona and **(b)** Texas, providing an early indicator of rapid acceleration in early June compared to mid-late May 2020. LRI is a daily reported ratio of the current week average of the percent positive PCR tests detected divided by the baseline percent in a county (1% in this example). LRI, local risk index; PCR, polymerase chain reaction.

Tracking of aggregate viral testing results from every employee's residential community may inform changes in the potential for exposure in home communities. Because approximately one third of the transmission of SARS-CoV-2 occurs in the communities where people live,^5^ local percent positive rates in the residential communities of each employee should be factored in. If employees commute to the office, they bring that residential exposure risk into common spaces. Residential community and close monitoring of trends (acceleration and deceleration) in positivity rates risk can be factored in by daily monitoring of the SARS-CoV-2 percent positive rates in those residential communities, as well as the graphic trends over time (Figure 1). The rate of acceleration can be measured by calculating the average percent positive rate of the *current* 7-day period divided by the average percent positive rate over the *previous* 7-day period.

3. Monitoring symptoms in the targeted population

Symptom monitoring (through temperature monitoring when entering the workplace and digital questionnaires) and enhanced testing are focused on employees who commute from areas with high percent positive rates or increasing trends over the past week (ie, a rapid rise in the proportion of positive results for SARS-CoV-2 among tested people).

4. Engaging people with COVID-19-like symptoms to enable contact tracing

Employees who become symptomatic or febrile should be identified immediately, isolated, and assessed as soon as possible with a viral PCR test. Their contacts also must be promptly engaged and assessed.

During early implementation of the above tactics, PCR testing via at-home self-collection to date has been offered to approximately 2000 Quest employees in geographic areas of higher pandemic burden (local risk index [LRI] >12.5%) in a voluntary testing program. Of approximately 400 tests processed, 4 (1%) had positive results. Employees with positive results were notified and began an isolation period. In addition, population testing inclusive of asymptomatic individuals appeared to escalate awareness and subsequently result in requested testing, as 4 additional employees submitted for illness-triggered testing.

## Data Integration and Surveillance

### Monitoring data in real time

To effectively suppress COVID-19 transmission in the workplace, employers can benefit from using real-time data to monitor employee health and inform action-alert decisions.^46^ An integrated system that pairs employee COVID-19 viral testing data and reported symptoms with employer/office health management and community data can facilitate the prompt identification of employees who are COVID-19 positive. The cloud-based platform from hc1 used by Quest Diagnostics organizes laboratory results from more than 2000 lab testing sites in the United States and more than 20 billion laboratory transactions representing more than 160 million Americans (half the US population). The data, including total tests and positive tests, feed specific customized tracking information for employers to measure and understand the local environmental risk factor of the percent positive viral PCR tests for SARS-CoV-2 down to the community level of their office location and the residential location of each employee. Data monitoring leverages a lab testing dashboard that was designed to track SARS-CoV-2 genome PCR testing results mapped for the 3007 counties in the United States and the numerous subcounty areas.

### Identifying community risk

To support administrative decisions regarding the best strategies to restrict or restart local activities, disease risk indices predict the risk of disease transmission in geographic regions.^47,48^ Epidemiological data can inform risk of contracting the virus through community spread.^49^ Risk-based surveillance enhances the ability to detect expected new cases as soon as possible by identifying those who are more likely to be infected than others.^50^ Insights into risk forecasting can be extracted from previous outbreaks^51,52^ and early views of the current pandemic.^47,48^

**FIG. 2.**
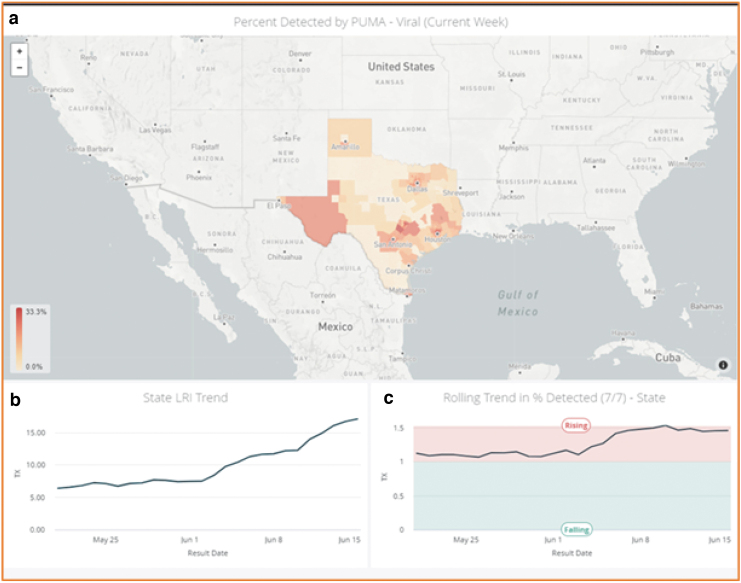
LRI showing epidemiological risk in Texas. **(a)** LRI dashboard for Texas subcounties (PUMAs): LRI is a daily reported ratio of the current week average percent positive PCR tests detected divided by the baseline percent in a county (defined as 1% in this example). **(b)** Statewide LRI trend over a 30-day period for Texas. **(c)** Rolling trend in the percent detected (7/7) – Texas: This is the average percent positive rate of PCR tests in the *current* 7-day period divided by the average percent positive rate over the *previous* 7-day period (abbreviated 7/7 or 7 vs 7). “Acceleration” is occurring if >1, and “deceleration” is occurring if <1. The rising 7/7 ratio (ie, >1) reflects the steady increase of LRI over the period of June 2 to June 15, 2020. LRI, local risk index; PCR, polymerase chain reaction; PUMAs, public use microdata units.

Using an LRI (daily reported ratio of the current week average percent positive PCR tests detected divided by the baseline percent in a county), epidemiological risk can be identified where an employee has an elevated chance of contracting the virus in that “hot zone” residential community when going about his/her activities of daily living (Figure 2). Community risk can be identified when the percent positive is significantly higher (eg, 10%–40%) than the desired (eg, <3%) epidemiological threshold at which local containment resources aimed at individual viral carriers alone would be enough to suppress a viral spread without mitigation efforts across the broader population of the community (LRI = 1.0).^53,54^ The Rockefeller Foundation has suggested thresholds of pandemic “mitigation” (percent positive <10) and “suppression” (percent positive <3).^55^ The higher the measured percent positive, the higher the LRI and the higher the statistical chance that an employee will be infected in her/his local community.^5,6^ The employee also would have a higher risk of contracting the virus from family members in the home environment, who are exposed to the higher prevalence in that same high-LRI community.

### Prediction of “hot spots”

Identifying individuals with COVID-19 while they are infected but are (as yet) unaware of their infection status is an objective of population-based “surveillance” testing^56^ for public health officials and for micropopulation-based “screening” in employer programming. Specifically, testing is implemented to engage employees with no or mild symptoms while they are shedding virus and, in doing so, create the opportunity for earlier isolation and mitigation of transmission to household members and coworkers. Targeting asymptomatic “screening” testing to individuals from areas with the highest COVID-19 burden, as demonstrated by larger population “surveillance” testing, should allow the highest yield while maintaining the greatest efficiency in the use of test kits, testing platforms, and related expenses.

As a leading indicator of population spread, LRI may predict rising community transmission (Figure 1). While death rates, ventilator rates, hospitalization rates, and emergency department visits are all lagging indicators of COVID-19 status, LRI (as an expression of percent positive) is the first objective leading indicator of local disease prevalence in a community. A rise in LRI can be detected weeks earlier than lagging indicators. As an example, after “mitigation orders” were first lifted in May 2020 in many states, people then flocked to Memorial Day holiday events that exposed tens of thousands to the COVID-19 virus.^57^ By July 4, lagging indicators such as numbers of emergency department visits, hospitalizations, and intensive care unit/ventilator transfers were accelerating (hc1 internal data representing the majority of 3007 counties in 50 states). Review of the statewide LRI tracking data tables of Arizona and Texas reflected a rapid acceleration in early June, indicating that these states were the hot spots to watch (Figure 1) well before the surge in lagging indicators.

Frequent monitoring of both the office location and residential locations of employees is key. LRI is updated daily and indicates the current level of active infection in an area and, by extension, the risk of contracting the virus for individuals in that area. An upward trend in the week-over-week ratio in LRI serves as an early warning that the prevalence of infection may be rising in an area. Potential employee interventions can include more frequent surveillance testing of employees from areas of upward trend and additional attention to tracking of data capturing employee self-reporting of symptoms. In contrast, a downward trend in LRI week-over-week ratio indicates that the level of infection is falling.

By regularly monitoring the latest LRI trends, employers have a better understanding of when and where to enhance broad community viral testing and use nonpharmaceutical interventions (eg, masks, distancing, handwashing) to confront the local impact of COVID-19. As businesses and universities reopen, employers can act with clear policies that include lab testing, symptom checks, and risk-aware plans based on LRI.

## Command Center

**FIG. 3.**
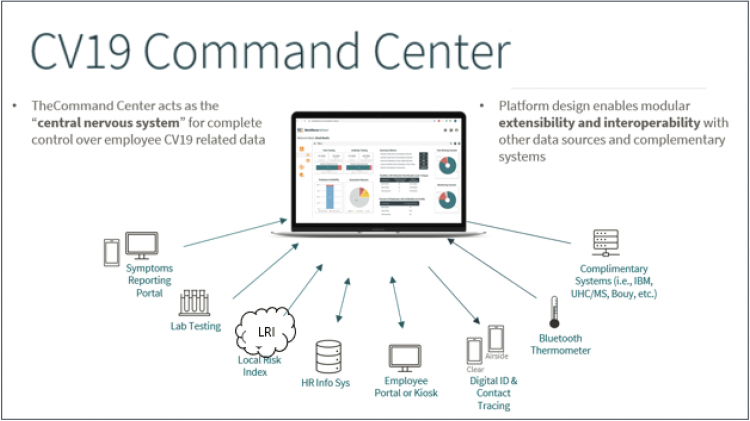
Workplace surveillance by a CV19 Central Command Center integrates internal and external data to inform decision-making. CV19, COVID-19.

The Command Center of a workforce COVID-19 surveillance program is the dashboard that brings together the network of data sources to facilitate decision-making and deployment of resources to essential business functions, by location, based on community risk, or in response to workplace exposure (Figure 3). It brings a network of data sources together across the whole employer population, down to the microgranular level of testing results and LRI risk of the individual employee. These data are automatically analyzed by embedded programming into “meaningful insights” that facilitate decision-making and deployment of resources to the most impactful areas of the business—essential functions, by location, based on community risk, or in response to workplace exposure. Command Center insights are graphically delivered exclusively to authorized users on HIPAA-compliant computers via secure encrypted internet linkage from the cloud-based data repository.

To create a comprehensive view of the risk of infection, the Command Center evaluates risk across 3 vectors: individual risk, local community risk (local infection rate), and workplace risk. Individual risk is calculated by evaluating the employee's response to symptom surveys and recent PCR test results. Local community risk is approximated by calculation of the LRI at the worksite and residential address at both the county and PUMA level. These factors are combined with the LRI of the workplace county to generate a workplace risk. This allows employers to evaluate the spread within the office, the surrounding community, and the neighborhoods of employees. A workplace index above 12.5% PCR positive rate per total tested may suggest the need for increased screening (2X–4X per month), contact tracing, and isolation from a given workplace.

To drive action, the Command Center analyzes each employee's symptom survey response and any test result as it is received, and the data can be leveraged by the company's Employee Health & Safety team to formulate an employee-specific action plan based on the specific scenario. Employees who declare they have illness symptoms and test positive for SARS-CoV-2 receive action alerts through the mobile health app directing them to isolate immediately. Employees who have no symptoms but report a high-risk contact with someone who has recently tested positive for SARS-CoV-2 also are directed, via their personal cell phone, to isolate for 14 days until it is documented they are no longer at risk for transmitting the virus. Trends and patterns of illness, isolation, and quarantine events can inform broader workplace actions (eg, need to transfer additional temporary staff to keep a location of operation section functional). Companies may wish to establish a threshold to trigger broader actions. For example, finding that 10% of a given work area has employees on isolation might trigger interventions such as more frequent workplace cleaning, asymptomatic testing, and contact testing, as well as possible preventive quarantine of additional employees from that work area.

### Digital ID application

The digital ID application provides a communication vehicle between the Command Center and the employee. The digital ID application is downloaded to each employee's cell phone or is available on their personal workstation. The Digital ID application will (1) accept individual SARS-CoV-2 test results in real time directly from the performing lab; (2) provide electronic verification of test results; (3) expedite capture of a “daily symptoms diary”; and (4) enable future contact tracing (optional, once nationally available) using a safe, secure, anonymized reporting engine.

As part of a digital ID solution, employee details (name, address, date of birth) are confirmed against an authoritative source from employment verification. These details are matched against any PHI before being included in the Digital ID. This ensures that test results are aligned with the identity of the individual.

## Conclusions

Widespread testing of populations for COVID-19, including employee populations with and without symptoms, can play a key role in identifying and isolating individuals with infection, thereby curbing further transmission. Integrating employee test information with population data into a real-time population health surveillance system may aid employer decision-making, policies, and practices to mitigate worker-to-worker transmission. Such an approach may fill an important gap in the collection of real-time data and the ability to convert those data into usable information (action alerts) in order to initiate appropriate localized public health actions in the microcommunity of the workplace. Follow-up analyses of the implementation strategy in different employer settings are required to determine the effectiveness of such efforts to mitigate person-to-person spread in the workplace.
